# Immobilization of *P. stutzeri* on Activated Carbons for Degradation of Hydrocarbons from Oil-in-Saltwater Emulsions

**DOI:** 10.3390/nano9040500

**Published:** 2019-04-01

**Authors:** Karol Zapata Acosta, Francisco Carrasco-Marin, Farid B. Cortés, Camilo A. Franco, Sergio H. Lopera, Benjamín A. Rojano

**Affiliations:** 1 Grupo de Investigación en Fenómenos de Superficie—Michael Polanyi, Departamento de Procesos y Energía, Facultad de Minas, Universidad Nacional de Colombia, Sede Medellín, Medellín 050034, Colombia; fbcortes@unal.edu.co (F.B.C.); caafrancoar@unal.edu.co (C.A.F.); 2 Grupo de Investigación Yacimientos de Hidrocarburos, Departamento de Procesos y Energía, Facultad de Minas, Universidad Nacional de Colombia, Sede Medellín, Medellín 050034, Colombia; shlopera@unal.edu.co; 3 Grupo de Investigación Materiales de Carbón, Departamento de Química Inorgánica, Facultad de Ciencias, Universidad de Granada, 18071 Granada, Spain; fmarin@ugr.es; 4 Grupo de Investigación Química de los Productos Naturales y los Alimentos, Facultad de Ciencias, Universidad Nacional de Colombia Sede Medellín, Medellín 050034, Colombia; brojano@unal.edu.co

**Keywords:** biomaterials, catalysis, crude oil, immobilization, hydrocarbons, produced water

## Abstract

Production water is the largest byproduct of the oil industry and must be treated before disposal, either by reinjection or shedding processes, with the purpose of eliminating emulsified crude oil and avoiding the operational and toxic problems associated with it. The objective of this work was to immobilize a hydrocarbon-degrading strain on activated carbons, to evaluate the biocomplex’s capacity for catalyzing hydrocarbons from Oil in Brine emulsions (O/W) simulating produced waters. Activated carbons were prepared and their chemical and porous properties were estimated by XPS, pH_PZC_ and SEM, N_2_ adsorption, and mercury porosimetry. Biomaterials were synthesized and hydrocarbon removal tests were performed. The basic and neutral carbons immobilized *Pseudomonas stutzeri* by physisorption in the macroporous space and electrostatic interactions (10^8^–10^9^ UFC∙g^−1^), while acid materials inhibited bacterial growth. Removal of aromatic hydrocarbons was more efficient using materials (60%–93%) and biomaterials (16%–84%) than using free *P. stutzeri* (1%–47%), and the removal efficiencies of crude oil were 22%, 48% and 37% for *P. stutzeri* and two biomaterials, respectively. The presence of minor hydrocarbons only when *P. stutzeri* was present confirmed the biotransformation process.

## 1. Introduction

One of the main products of the oil industry is production water, i.e., water that is produced along with the oil, and water that is injected during recovery processes. Production water is usually contaminated with hydrocarbons, which must be removed before either reinjection or disposal [1]. For each barrel of oil, approximately five barrels of water are produced, and, in mature fields, this ratio could reach 1:15 [2]. This situation has required the development of technologies to ensure control of the production water in accordance with industry standards for its disposal or reinjection (National Association of Corrosion Engineers NACE). Currently, there are different mechanisms for the removal of oil from the production water including adsorption [3], filtration [4], reverse osmosis [5], flotation [6] and coagulation [7]. Adsorption is the most commonly employed technique in the tertiary stage of oil removal (“dissolved” oil) due to its simplicity and effectiveness. Typical adsorbents used in the treatment of produced water are activated carbon, organoclay, copolymers, aerogels, zeolites, resins and, lately, nanomaterials. 

The combination of absorbents as carbons and organoclays is efficient for total oil removal (TPH) up to 85% [8], and the use of nanomaterials in hydrocarbon adsorption processes in the aqueous phase has been 100% efficient [9]. Regardless of the positive results, the adsorptive approaches have limitations for the desorption and final elimination of the contaminant. Biological processes are an emerging technology capable of solving these issues due to the capacity of specific microorganisms to catalyze “miscible” hydrocarbons in water [10]. While the physical treatments and many of the chemicals are based on transferring the contamination between gaseous, liquid and solid media, in the biological processes the contaminating compound is transformed into another more oxidized and less toxic state. 

Likewise, biological developments are less invasive methods and generally do not require the structural or mechanical components that pose a threat to the environment. Biological processes are also cost-effective and, because they are natural, are usually accepted by the public [10,11,12]. Some researchers have demonstrated the early disappearance of microbial consortium introduced in an environment contaminated with hydrocarbons [13,14].

The reason is that there are stress factors, high temperatures, water activity, pH, dissolved oxygen content, nutritional limitations, and toxicity of contaminants, which limit cell viability. Consequently, the development of efficient biological technologies for the removal of hydrocarbons is of great ecological and economic value. Strategies such as the immobilization of microbial cells in materials are being used [15].

The support for microbial immobilization provides a niche in microbial inoculants through the provision of a protective surface or porous space. The contaminants are first absorbed on the surface of the material in which the microorganism is immobilized and then gradually pass through the pores; the participation of these three types of adsorbates generate a biofilm that favors the transformation of the contaminant [16]. 

Therefore, organisms have sufficient contact time for their catalytic action. Several synthetic polymers, polyacrylamide, polyethylene glycol and polyurethane [17,18], carrageenan, agar, collagen, chitin and chitosan [19], have been used as matrices for the immobilization of microorganisms. They have also been used as a wide range of carriers prepared from natural materials, clays [16], activated carbons [20], and other materials [11,21,22].

However, to the best of our knowledge, no research has yet been conducted on the immobilization of microorganisms on activated carbon for the removal of hydrocarbons in O/W emulsified systems. Activated carbons are structured materials with high porosity and large surface chemistry, controllably obtained from the pyrolysis of organic matter, which can be used as important adsorbent materials of bacteria and hydrocarbons [23,24,25]. 

This research aims to understand the effects of immobilizing hydrocarbon-degrading bacteria on activated carbon and evaluating the biomaterial capacity to remove aromatic hydrocarbons and crude oil from simulated produced water. 

## 2. Materials and Methods 

### 2.1. Materials 

Olive stones were harvested in Granada, Spain, and were used as the raw material for the activated carbon preparation. The olive stones were dried at 80 °C until constant weight and sieved to 1.6–2.4 mm size. Potassium hydroxide (reagent grade ≥90%, Sigma-Aldrich, St. Louis, MO, USA) and phosphoric acid (mass fraction of 85% in H_2_O, Sigma-Aldrich, St. Louis, MO, USA) were used for the chemical activation of the olive stones before carbonization, while melamine (99%, Sigma Aldrich, St. Louis, MO, USA) and phosphoric acid (mass fraction ≥85% in H_2_O, Sigma-Aldrich, St. Louis, MO, USA) were used for the modification of surface chemistry. Chlorihydric acid was used for the washing protocol (mass fraction of 37% in H_2_O, Sigma-Aldrich, St. Louis, MO, USA). Acetonitrile (mass fraction of 99.8% in water, Sigma-Aldrich, St. Louis, MO, USA) was employed for rhamnolipid quantification. Tryptone glucose extract agar (TGEA) and bushnell haas broth (BH, Sigma Aldrich, St. Louis, MO, USA) were used for the bacterial culture. A Colombian light crude oil (33° API), deionized water and NaCl (99%, Merck KGaA, Darmstadt, DE, Germany) were used for preparing oil in brine emulsions. Benzene, toluene, and phenol (≥98%, CARLO ERBA Reactive-SDS, Barcelona, ES, Spain) were used for the assays of the removal of aromatics hydrocarbons.

### 2.2. Methods

#### 2.2.1. Preparation of Activated Carbons

Activated carbons were prepared by chemical activation of olive stones with phosphoric acid (CP) and potassium hydroxide (CK) following the procedure previously published by Bautista et al. [26]. A portion of olive stones was combined separately with potassium hydroxide and phosphoric acid in a mass ratio of 1:2. The blend was macerated and dried under IR lamps. It was then carbonized by heating at 10 °C·min^−1^ to 840 °C with a soak time of 2 h under a nitrogen flow of 300 cm^3^·min^−1^ using a tubular furnace (Heraeus, Hanau, Germany). The CK was washed with HCl 0.1 N and ultrapure water until no chloride ions were present in the washing water. The CP was cleaned with distilled water until pH = 7.

#### 2.2.2. Chemical Modification of Activated Carbons

An incipient impregnation method was used for surface modifications [23]. The CK and CP materials were dried at 110 °C for 24 h. An appropriate amount of modifying agents, phosphoric acid and melamine were prepared with a minimum amount of water or ethanol, according to their solubility for including heteroatoms of P or N, respectively. The solutions were then slowly dripped on each material. Impregnated materials were dried under IR lamp for 24 h and were later heated at 700 °C for 1 h under a nitrogen flow of 300 cm^3^·min^−1^ using a tubular furnace (Heraeus). Finally, four materials were synthesized, CK and CP with melamine, labeled as CKN and CPN, respectively, and CK and CP with phosphoric acid, to obtain CKP and CPP, respectively. 

#### 2.2.3. Characterization of Activated Carbons

The material morphology was studied by scanning electronic microscopy (SEM) using a LEO (Carl Zeiss, Oberkochen, Germany) GEMINI-1530 microscope. Textural characterization was carried out by N_2_ adsorption at −196 °C using Quadrasorb SI equipment (Quantachrome instruments, Boynton beach, FL, USA). Prior, the carbon samples were degassed overnight at 100 °C. The Brunauer–Emmett–Teller (BET) equation was applied to the N_2_-adsorption isotherms to determine the surface area (S_BET_). The Dubinin–Radushkevich equation was used to determine the micropore volume (V_mic_) and the mean micropore width (L_mic_). Furthermore, the Barrette-Joynere-Halenda (BJH) method was used to calculate the mesopore volume (V_meso_) and the mean mesopore width (L_meso_). The total pore volume (V_0.95_) was considered to be the volume of N_2_ adsorbed at P/P_0_ = 0.95 [23]. Mercury porosimetry was carried out using Micromeritics (Norcross, GA, USA) AutoPore IV 9510 equipment up to a pressure of 60,000 psi to obtain the mesopore volume V_2_ (dpore 6 to 50 nm), macropore volume V_3_ (dpore 50 to 10,000 nm) and mean macropore width (L_macro_). Pore size distributions (PSD) were obtained by coupling the BJH and mercury porosimetry results. The material chemical characterization was analyzed by X-ray photoelectron spectroscopy (XPS), and information about the material acidity was obtained by measuring the pH_PZC_, according to the methodology described by Perez et al. [26]. 

#### 2.2.4. Immobilization Kinetics

The method proposed by Sekaran et al. [20], with some modifications, was employed. For this, 0.01 g of sterile materials were mixed with 5 mL of *P. stutzeri* (previously isolated and identified) and suspended (10^10^ u.f.c) in NaCl 0.9% and a mass fraction of 0.33% of BH®. The mixture was shaken at 100 rpm and 35 °C. Every three hours, the material was retired and washed with K_2_HPO_4_ 0.15 M to eliminate weakly adhered cells. Lastly, the number of cells on the material was analyzed by plate count in Tryptone Glucose Extract Agar (48 h/35 °C). The results were expressed as the number of cells immobilized per gram of material. 

Carbon sterilization was carried out following the method described by Sekaran et al. [20]. For this, approximately 1 g of carbon was placed in an Erlenmeyer flask of 200 mL, slightly closed, and autoclaved (WestLab, Surrey, UK) at 121 °C and 15 psi for 15 min. The manipulation of the materials was always carried out in a laminar flow chamber (DSS, New Delhi) to guarantee asepsis. Sterilization was based on the denaturation of the proteins of the microorganisms due to the moist heat supplied by the autoclave.

#### 2.2.5. Preparation of Oil-Brine Emulsions (O/W)

Saltwater (brine) was prepared by mixing NaCl and deionized water to obtain a mass fraction of 2% in H_2_O, based on a procedure previously reported by our group [27]. Oil in brine (O/W) emulsions were prepared by mixing 8000 mg·L^−1^ of crude oil and brine with a mass fraction of 0.33% Bushnell Haas Broth BH^®^ (nitrogen, phosphates and minerals source) at 10,000 rpm for 20 min. The stability of the emulsions was monitored for 120 h by observing the size of the oil drops, using an optical microscope, and the changes in absorbance, using UV-VIS spectrophotometer Genesys 10S (Thermo Scientific, Massachusetts, US) [28]. In parallel, brines with specific hydrocarbons were prepared by mixing benzene, toluene and phenol 600 mg·L^−1^ at 600 rpm for 10 min in deionized water with a mass fraction of 0.33% of Bushnell Haas Broth BH^®^.

#### 2.2.6. Hydrocarbon Degradability Test

Crude oil and aromatic hydrocarbon degradability tests were performed following the protocol proposed by Juang and Tsai [29], with some modifications. The biomaterials were inoculated into 15 mL of W/O emulsions. After, the flasks were cultured at 150 rpm and 35 °C for 24 h. Free cells (10^6^ u.f.c) were used as a control. Crude oil and aromatic hydrocarbon content was analyzed by Gas chromatography–mass spectrometry (GC-MS) and High performance liquid chromatography (HPLC), respectively, according to the method reported by Galazka [30].

After the isolation and identification of *P. stutzeri*, the biomass was obtained according to the method described by Juan and Tsai [29]. For this, the pure culture of *P. stutzeri* was cultured in Nutrient Broth (Sigma-Aldrich, St. Louis, MO, USA) for 60 h at 150 rpm and 35 °C, until it reached the stationary state. Then, the biomass was filtered and stored under asepsis.

#### 2.2.7. Rhamnolipid Quantification 

For the rhamnolipid quantification, standard solutions were prepared (CAS Standard: 0869062420 Sigma-Aldrich, St. Louis, MO, USA) and the calibration curve was elaborated using HPLC (Prominence, Shimadzu, Kioto, Japan). A LiChrospher^®^ 100 RP-18 column (5 µm; 250 × 4 mm) was used as the stationary phase and water/acetonitrile 1:1 as the mobile phase. The experimental conditions were an absorption wavelength of 254 nm, an injection volume of 70 µL, 27 °C of temperature, a flow rate of 0.8 mL·min^−1^ and a retention time of 2.5 min.

#### 2.2.8. Statistical Analysis

Immobilization kinetics and bio-removal experiments were made in triplicate. The reported data correspond to the arithmetic mean ± standard deviation. In all cases, deviations (±SD) were lower than 10% of the mean (coefficient of variation). To determine statistical differences between measurements, a unidirectional analysis of variance was performed in the 95% confidence interval using Statgraphics Centurion V (Statgraphics, Seattle, WA, USA). Comparisons of the means were made with a Duncan’s multiple range test (*p* < 0.05).

## 3. Results and Discussion

### 3.1. Characteristics of Activated Carbons

**Figure 1:**
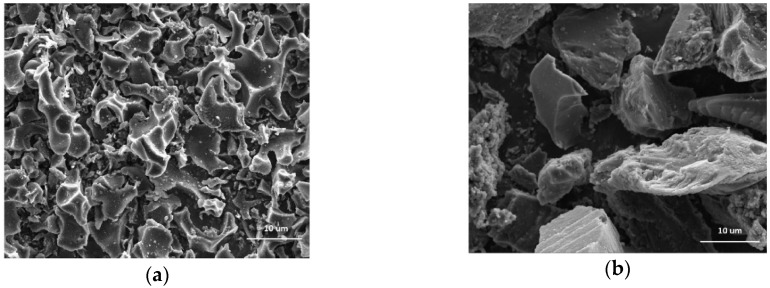
The microstructure of activated carbons prepared by chemical activation of olive stones with (**a**) potassium hydroxide (CK) and (**b**) phosphoric acid (CP); estimated by SEM analysis.

The morphologies of (a) CK and (b) CP samples are shown in Figure 1. CK and CP are a three-dimensional network of amorphous particles. CP, in particular, presents bigger and wider units. Visually, the CK pores are smaller than CP, which is in agreement with the results reported by Moreno et al. [31]. Table 1 summarizes the chemical composition of CK and CP samples. Overall, the surfaces of the materials are constituted mainly of C (>85%) and O (7.7%) atoms. The CP material showed residual phosphorus due to the H_3_PO_4_ employed for activation (mass fraction of 6.7%).

The specific chemical bonds in the surfaces of CK and CP were assessed by deconvolution of XPS signals, as shown in Table 2. The carbon spectra showed six peaks for CK and CP related to carbon atoms bonded to carbon and oxygen. The C=C bound the most frequently (≥70% of carbon bonds) for both samples were C=C, C–O, C=O, O=C–OR, $CO_{3}^{-2}$ and $CO_{2}$. Studying the Oxygen spectrum, CK and CP presented two peaks, which corresponded to the presence of i) double bonded oxygen, O=C and/or O=P (50% oxygen bonds), and ii) single bonded oxygen, O–C and/or P–O–C [32,33,34]. Regarding the P_2_p region of the CP sample, three different peaks were found: reduced phosphorus (C–P; 40% phosphorous bonds), Pyrophosphate (C–PO_3_; 40% phosphorous bonds), and polyphosphates and/or phosphates (C–O–PO_3_; 20% phosphorous bonds). Hasegawa et al. [35] observed that P atoms are introduced into carbon principally in reduced states and these unstable groups are gradually oxidized by oxygen, leading to oxidized P-containing functional groups. In the CP sample, the 60% phosphorus group was oxidized.

**Figure 2:**
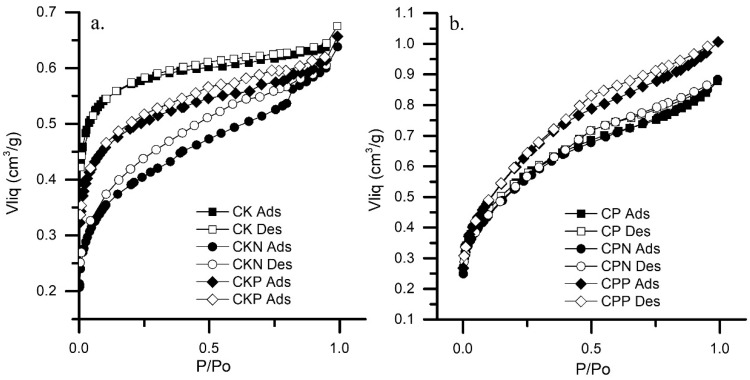
Nitrogen isotherms of activated carbons prepared by the chemical activation of olive stones with (**a**) potassium hydroxide (CK) and (**b**) phosphoric acid (CP). Adsorption curve—filled symbols; desorption curve—unfilled symbols.

All isotherms are represented in Figure 2. The results show that the CK isotherm was type I, while the other materials exhibited type II isotherms. The CK isotherm was concave in terms of the relative pressure axis (P/P_0_), increased rapidly at low pressures (P/P_0_ < 1 × 10^−3^) and later reached horizontal saturation. This class of isotherm is characteristic of highly microporous materials. The high adsorption energy of the micropores allows the gas to adsorb at low pressures. Once the entire volume of the micropores has been completed, the curve remains almost constant over a wide range of pressures, producing the so-called plateau.

CKN, CKP, CP, CPN and CPP isotherms were also concave at low pressures (P/P_0_ < 1 × 10^−3^) with respect to the relative pressure axis (P/P_0_) but later increased linearly and finally became convex. This phenomenon assumes monolayer adsorption at low pressures (micropores), as well the formation of multimolecular layers at medium and high ranges of relative pressures (mesopores) [36]

The material’s textural properties are summarized in Table 3. CK materials were exclusively microporous, their micropore volume (V_micro_) represented approximately 80% of V_0.95_, while CP materials were micro and mesoporous; V_micro_ and V_meso_ each represented 50% of V_0.95_. The CP samples exhibited a significantly higher V_0.95_ than the CK samples. The difference between mesoporous volume by BHJ method (V_meso_) and mesoporous volume by porosimetry method (V_2_) indicated that, for all materials, the mesopores were between 2 and 6.5 nm. 

The surface area (S_BET_) for CK and CP decreased with the surface modifications, which was to be expected considering that the heteroatoms block the pore by deposition in the microporous space. However, the modification with phosphoric acid (P) of CP had a double activating effect, increasing V_micro_ and S_BET_.

**Figure 3:**
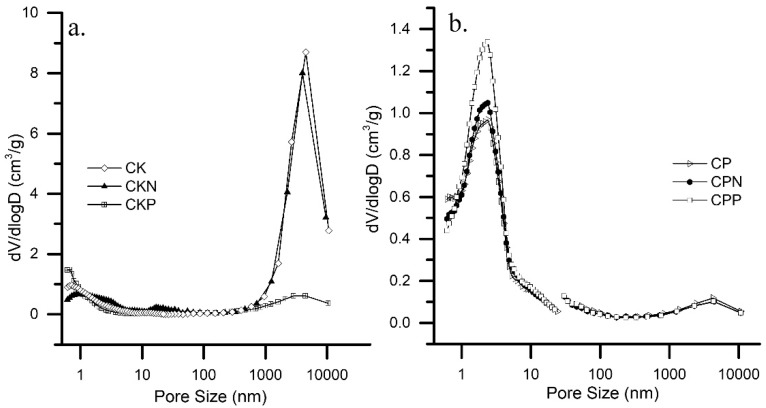
Pore size distribution of activated carbons prepared by chemical activation of olive stones with (**a**) potassium hydroxide (CK) and (**b**) phosphoric acid (CP) functionalized with melamine (CKN and CPN), and phosphoric acid (CKP and CPP).

V_3_ obtained by mercury porosimetry showed that CK samples, besides being micro, were prominently macroporous materials, especially CK and CKN, and confirmed that CP samples were exclusively micro and mesoporous materials. Pore size distributions (PSD) are plotted in Figure 3. 

Both distributions showed multimodal behaviors; the mean pore width in the CK samples was centered on *d*, 1.03 ± 0.41 nm and 4533 ± 293 nm, with final percentages of micro and macroporosity of 18% and 76%, respectively. CP samples exhibited a mean pore width centered on *d*, 2.25 ± 0.11 nm and 4342 ± 91 nm, with final percentages of meso and macroporosity 50% and 12%, respectively. 

CK samples exhibited significant macropore volume because of the large space located between the fine primary particles (low bulk density), unlike the CP samples that had less interparticle space (high bulk density); similar results were previously reported for microporous carbons [20,37]. 

The pH_PZC_ values are also included in Table 3. All CP materials were strong acids due to the use of acid activation that promoted cleavage reactions and the formation of crosslinks with the phosphorous atom (Lewis acids), as verified by XPS. CK and CKP materials were slightly acidic or almost neutral, while CKN was a basic material for the presence of amino groups on the surface (Lewis basis); similar data were obtained by Moreno [38].

### 3.2. Immobilization Kinetics 

**Figure 4:**
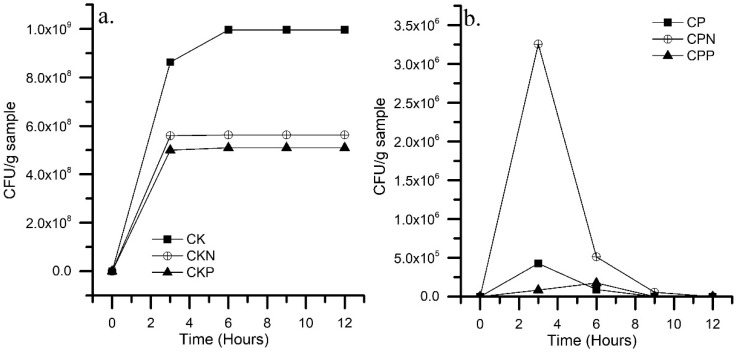
Immobilization kinetics of *P. stutzeri* on activated carbons prepared by chemical activation of olive stones with (**a**) potassium hydroxide (CK) and (**b**) phosphoric acid (CP); functionalized with melamine (CKN and CPN) and phosphoric acid (CKP and CPP).

The immobilization kinetics of *P. stutzeri* on CK and CP materials are shown in Figure 4. The results indicate that for all CK materials the immobilization process advanced linearly with the interaction time until the third hour and equilibrium was reached after. The linear zone was attributed to the migration of the cells from the bulk cell suspension to the particle surfaces, while the plateau region corresponded to the diffusion of *P. stutzeri* from the surface to the interior of the pores [20]. 

The ability to immobilize cells is given in the following order: CK > CKN > CKP. The results were based in the bacterial–material interaction. The cationic and neutral surface of CKN and CK under experimental pH attracted the negative charge of the cell wall. Both Gram-negative and positive bacteria possessed a relatively negative charge on their surface due to the presence of active proton functional groups, such as hydroxyl, carboxyl, and phosphoryl deprotonated to pH = 7.0 [39]. CK and CKN materials exhibited remarkable macroporous volume where bacteria could be hosted. Similar results were previously reported [20].

In CP materials, after 6 h of contact, no bacterial cells remained attached to the materials. The results can be explained by considering that the CP materials exhibited low pH_PZC_ and were very negative in neutral media, presenting strong repulsions with the bacterial exocellular membrane that is also negatively charged under the same conditions. In addition to the low adsorption of microorganisms, the absence of a bacterial count in the final suspension established the antimicrobial effect of CP materials. When acid materials were exposed to a pH of 7 they were negatively charged due to the hydrogen release that came from the dissociation of the surface chemical groups such as hydroxyl and carboxyl [39]. Subsequently, the proton concentration in the medium increased and the pH was reduced, leading to the antimicrobial effect. The acid media affected the bacterial membrane H^+^-ATPase and denaturalized the bacterial enzymes, causing cell death.

**Figure 5:**
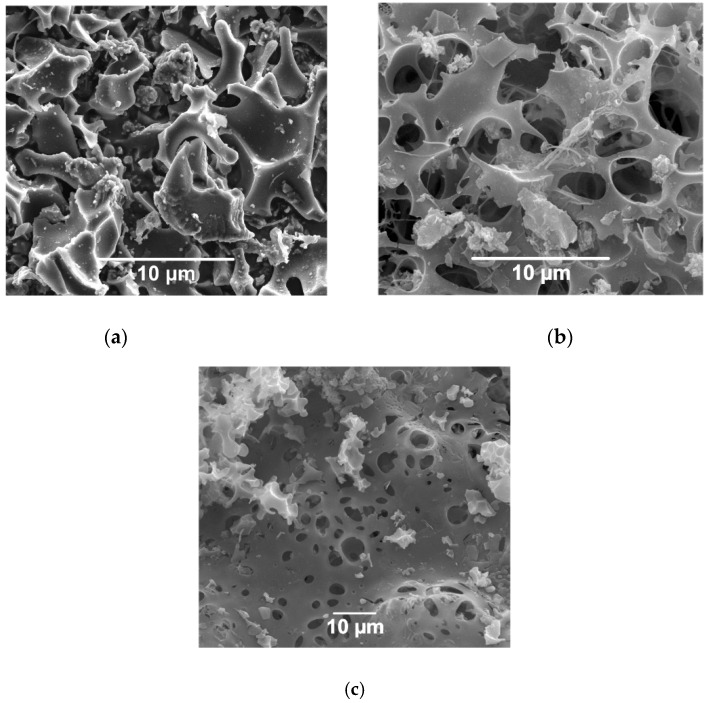
The microstructure of activated carbons prepared by chemical activation of olive stones with potassium hydroxide (CK), (**a**) without *P. stutzeri*, (**b**) *P. stutzeri* on CK surface, and (**c**) *P. stutzeri* on melamine-functionalized CK (CKN); surface estimated by SEM analysis.

CK and CKN were chosen as suitable materials for the immobilization of *P. stutzeri*. The cells adsorbed on CK and CKN were 1.0 × 10^9^ and 6.6 × 10^8^ UFC·g^−1^, respectively (biomaterials). The results of this investigation are in accordance with other reports [40]. The SEM micrographs confirmed biomaterial formation, as shown in Figure 5. The presence of *P. stutzeri* on the materials was evidenced by the formation of a continuous layer (biofilm) that was absent in CK materials. 

### 3.3. Hydrocarbon Degradability Test

**Figure 6:**
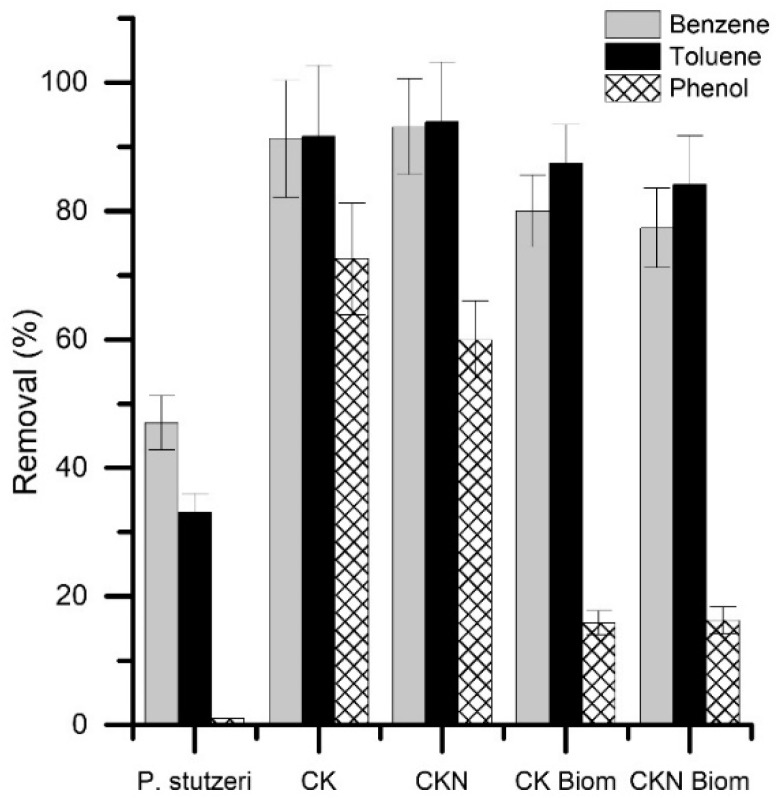
Aromatic hydrocarbon removal by activated carbons prepared by chemical activation of olive stones with potassium hydroxide (CK), functionalized with melamine (CKN), *P. stutzeri* on CK (CK Biom.) and CKN (CKN Biom.) materials.

The biomaterial’s capacity to remove aromatic hydrocarbons from oil-brine emulsions (O/W) is displayed in the Figure 6. *P. stutzeri*, CK and CKN materials were used as the controls. In particular, the adsorption efficiencies of benzene and toluene, using CK and CKN materials, were greater than 90%, while their capacity to adsorb phenol was less than 70%. These results can be explained by considering the difference in polarity between the adsorbates. The capacity of *P. stutzeri* to metabolize aromatic hydrocarbons can be understood as follows: benzene > toluene > phenol. *P. stutzeri’s* ability to consume phenol from a simulated medium was almost nonexistent. 

In particular, the adsorption efficiencies of benzene and toluene using CK and CKN materials, were greater than 90%, while their capacity to adsorb phenol was less than 70%; these results can be explained by considering the difference in polarity between the adsorbates. The capacity of *P. stutzeri* to metabolize aromatic hydrocarbons is as follows: benzene > toluene > phenol. *P. stutzeri’s* ability to consume phenol from a simulated medium was almost nonexistent. 

The use of CK and CKN biomaterials for phenol removal produced a drop in the removal efficiency, originally greater than 50%, of CK and CKN materials. These results could be attributed to the loss of porosity when *P. stutzeri* was immobilized, as well as the absence of any *P. stutzeri* affinity for phenol. The experiments in the presence of phenol were useful to interpret the ways in which the cells altered the porosity of CK and CKN. The comparison between the phenol removal efficiencies using materials and biomaterials shows the way the pore space was modified by bacterial immobilization. Due to the low ability of *P. stutzeri* to consume phenol, the removal was strictly limited to the adsorptive process. 

The use of CK and CKN biomaterials for benzene and toluene elimination caused a less than 15% reduction in the removal efficiency of CK and CKN materials. These results suggest that the loss of active sites by bacterial immobilization was compensated for with *P. stutzeri’s* ability to consume benzene and toluene individually. Finally, there are no significant differences between CK and CKN, or CK and CKN biomaterials in terms of aromatic hydrocarbon removal. 

**Figure 7:**
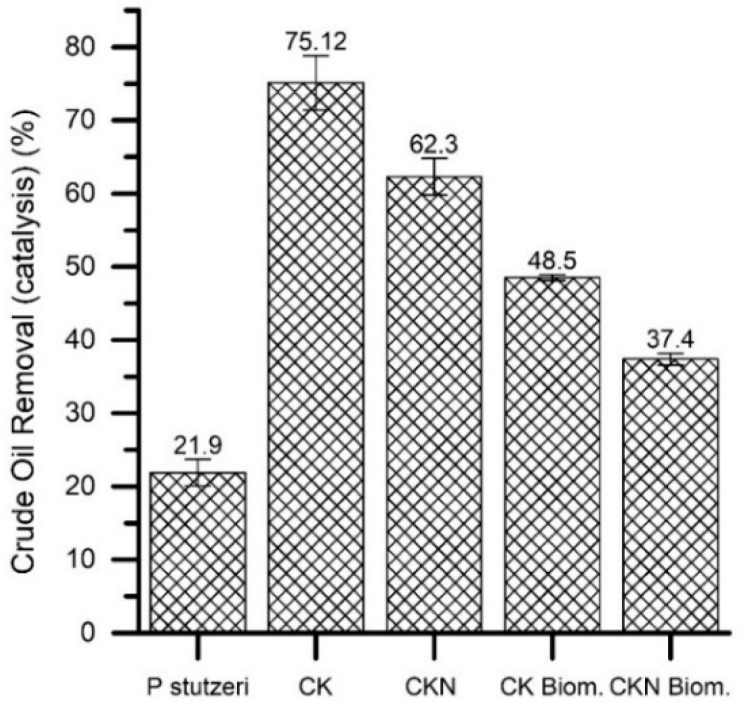
Crude oil removal by activated carbons prepared by chemical activation of olive stones with potassium hydroxide (CK) with and without functionalization with melamine (CKN) and by biomaterials of *P. stutzeri* on CK (CK Biom.) and CKN (CKN Biom.).

Briefly, the removal of aromatic hydrocarbons was more efficient using materials (60%–93%) and biomaterials (16%–84%) than free *P. stutzeri* (1%–47%). The adsorption was favored by the material’s porosity and adsorptive couple polarity. CK and CKN displayed a high degree of microporosity, in which benzene, toluene, and phenol, with molecular volumes around 100 Ǻ, could be accommodated. The non-polar character of CK and CKN guaranteed the hydrophobic attraction of the hydrocarbons, also apolar. The CKN cationic surface favored aromatic hydrocarbon retention since the π electrons in the aromatic ring were electrostatically linked with the surface cations. Other authors have reported the high affinity of this adsorptive couple [41,42]. Crude oil removal capacity using CK, CKN and biomaterials are shown in Figure 7. Free *P. stutzeri* and oil-brine emulsions (O/W) were used as controls. 

The total crude oil removed from the oil-brine emulsions (O/W) was, after 24 h, 21.9%, 75.1%, 62.3%, 48.5% and 37.4% using, respectively, *P. stutzeri*, CK, CKN, CK and CKN biomaterials. In general, CK and CKN materials removed a higher amount of crude oil than the biomaterials. This could be due to the high physicochemical affinity of the adsorptive couple that could be ruled by the Van der Waals interactions. The high porosity of the carbons benefits the physisorption of the crude oil components. The difference between the removal of the base materials and the biomaterials lies in the different amounts of available active sites, which are less in the biomaterials since the microorganisms are deposited in the pores. CKN showed the best result (48.5% of crude oil removed from 8000 mg·L^−1^ in OBE after 24 h); these findings are explained by considering that the CK material immobilized *P. stutzeri* in greater proportion and presented better porous properties. 

**Figure 8:**
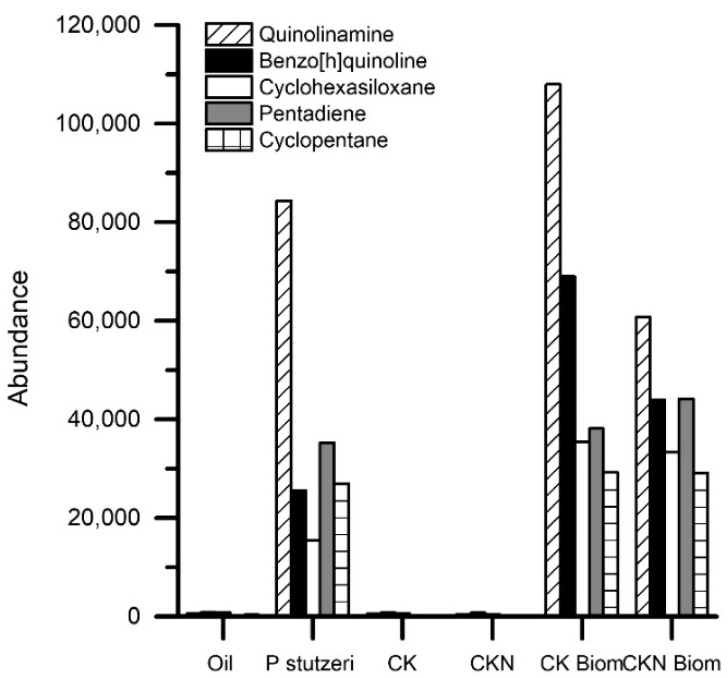
Biocatalysis products from crude oil removal by activated carbons prepared by chemical activation of olive stones with potassium hydroxide (CK), functionalized with melamine (CKN), *P. stutzeri* on CK (CK Biom.) and CKN (CKN Biom.) materials estimated by GC-MS analysis.

Although there is evidence of oil removal efficiencies higher than those reached in the present study (≥90% removal in less than 24 h), these results were obtained with adsorbent and non-catalytic materials so the advantages are partial. They do not solve the problems of desorption, such as in the present work in which biomaterials are used. In this study, the catalytic action of the biomaterials was confirmed as the presence of minor hydrocarbons, such as quinolinamine, benzo[h]quinoline, cyclohexasiloxane, pentadiene, and cyclopentane, which were observed only in the presence of free or immobilized *P. stutzeri*, unlike remediation with CK and CKN base materials (Figure 8). 

A biochemical character that the authors have systematically examined is the mechanism of hydrocarbon catalysis by the *Pseudomonas* genus. Biocatalysis was described as a process that culminates in the cleavage of the aromatic rings and the final formation of fatty acids and/or acetyl CoA for the development of pathways to obtain gas and water. During the first stages of catalysis, the addition of oxygenated groups, specifically hydroxyls (Monooxygenases), and the subsequent oxidation of these groups to aldehydes, ketones and carboxylic acids (Dehydrogenases) was reported [43,44]. In the present study this phenomenon was evidenced by the presence of homologous metabolites of benzene, which were modified by the addition of functional groups of oxygen, hydroxyl, carboxyl, ester, and ether. 

Finally, existing reports suggest that the degradation efficiency is largely determined by the degree of retention of contaminants (which is governed by the process of adsorption, desorption, and diffusion between the porous materials). In other words, the contaminant’s availability is a critical factor of hydrocarbon biocatalysis [45,46]. 

Consequently, the bioremediation capacity is related to the strain’s ability to access the adsorbed hydrocarbon. It is therefore believed that degrading hydrocarbon strains produce surfactants for this purpose. During xenobiotic bioremediation modulated by fine porous materials, some types of adsorption phenomena may take place: the contaminant’s adsorption on the material, the cell’s adsorption on the material and the contaminant’s adsorption on the microbial cells. These adsorption phases lead to the formation of a microenvironment or biofilm for pollutant degradation [47]. 

However, contaminants can be blocked within the micro/mesopores of the materials. Due to the size factor, a bacterium cannot enter these spaces, causing the hydrocarbons to be blocked to microbial action. It is hypothesized that degrading hydrocarbon strains produce biosurfactants that decrease the surface tension that occurs in all phases [48]. 

**Figure 9:**
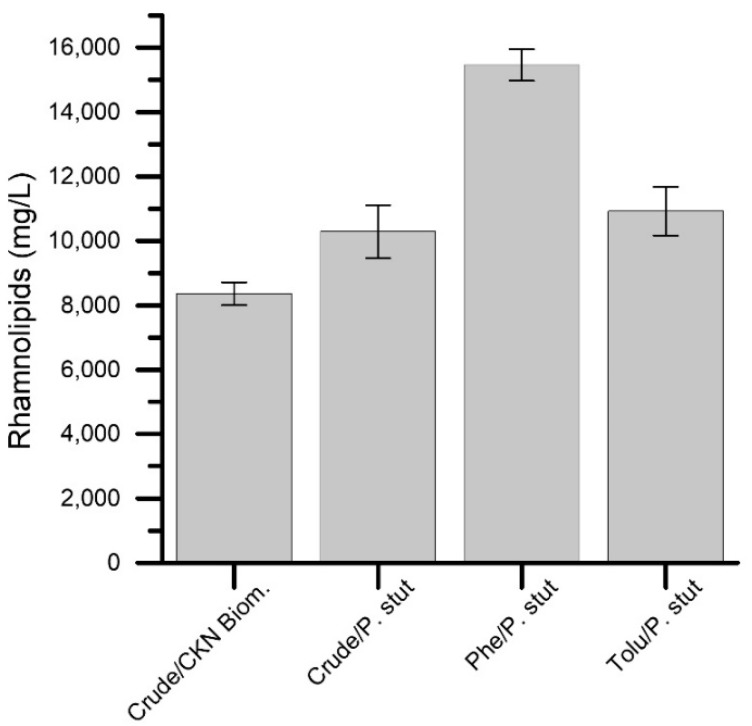
Rhamnolipid production by *P. stutzeri* and *P. stutzeri* on CKN after biocatalysis of aromatic hydrocarbons and crude oil.

Figure 9 shows the rhamnolipid production by *P. stutzeri* and *P. stutzeri* on CKN (the best biomaterial) during the aromatic hydrocarbon and crude oil removal; the results confirmed the biosurfactant generation for bioremediation action. Rhamnolipid production in the presence of phenol is remarkable; the results could be related to the difficulty for *P. stutzeri* to metabolize this hydrocarbon exclusively. This condition can force the production of surfactants by the microbial cell. Many authors have reported the rhamnolipid production by strains of the genus *Pseudomona* for the effective removal of hydrocarbons (10–20 g/L) similar to those found in this study (9 g/L–16 g/L) [49,50]. On the other hand, the production of rhamnolipids when *P. stutzeri* was immobilized on CKN (CKN Biomaterial) was less efficient compared to the other tests, which could be due to the partial adsorption of the biosurfactant in the porous material.

## 4. Conclusions

This study investigated the capacity of biofilm activated carbon *P. stutzeri* to catalyze hydrocarbons and crude oil from oil-in-saltwater emulsions. The results showed that the macroporosity of the materials was essential to bacterial immobilization, the microporosity for the hosting of the hydrocarbons, and high pH_PZC_ for the electrostatic stabilization of these adsorbates. The removal of aromatic hydrocarbons was slightly more efficient using materials (adsorption: 60%–93%) and biomaterials (catalysis: 16%–84%) as opposed to free *P. stutzeri* (bioremediation: 1–47%). Total crude oil removal from OBE systems using *P. stutzeri*, CK, CKN, CK and CKN biomaterials was 21.9%, 75.1%, 62.3%, 48.5% and 37.4% after 24 h. The lower removal percentages, compared with purely adsorptive methods, are compensated for with the catalytic route for the oil elimination. This is evidenced by the presence of minor hydrocarbons only in the presence of free or immobilized *P. stutzeri*. Future works should validate more extensive biodegradation kinetics.

To the best of our knowledge, this is the first research aimed at the development of biocatalysts to remove crude oil from produced water, for use in offshore technologies. 

## Figures and Tables

**Table 1:** Chemical composition of activated carbons prepared by chemical activation of olive stones with potassium hydroxide (CK) and phosphoric acid (CP); estimated by XPS analysis.

Sample	C	O	P
	(Mass Fraction %)	(Mass Fraction %)	(Mass Fraction %)
CK	92.3	7.7	-
CP	85.5	7.7	6.7

**Table 2:** Chemical bonds of activated carbons prepared by chemical activation of olive stones with potassium hydroxide (CK) and phosphoric acid (CP); estimated by deconvolution of XPS signals.

Peak	Binding Energy	Assignment	% Peak	
			CK	CP
O1s	531.3	C=O/P=O*	48	40
	533.0	C-O/C-O-P-O/C-PO*	52	60
C1s	284.5–284.6	C=C/C-O-P*	73	72
	285.5–286.4	C-O	17	17
	287.4–287.6	C=O	4	5
	288.4–288.48	O=C-OR	3	4
	290.4–290.8	$CO_{3}^{-}$ $CO_{2}$	4	2
P2p	132.3	Reduced phosphorus compound C-P (P-1)	-	39
	133.1	Pyrophosphate, C-PO3 (P-2)	-	39
	134.1	Polyphosphates and/or phosphates, C–O–PO3 (P-3)	-	22

**Table 3:** Textural proprieties and point of zero charge (pH_PZC_) of activated carbons prepared by chemical activation of olive stones with potassium hydroxide (CK) and phosphoric acid (CP).

Sample	pH_PZC_	Bulk Density	S_BET_	N_2_-ADSORPTION					MERCURY POROSIMETRY		
				V_micro_ (0.5–2 nm)	L_micro_	V_meso_ (2–30 nm)	L_meso_	V_0.95_	V_2_ (6.5–50 nm)	V_3_ (50–10,000 nm)	L_macro_
		g·cm^−3^	m^2^·g^−1^	cm^3^·g^−1^	nm	cm^3^·g^−1^	nm	cm^3^·g^−1^	cm^3^·g^−1^	cm^3^·g^−1^	nm
CK	6.69	0.113	1395	0.496	1.48	0.119	2.20	0.638	0.054	4.906	4456
CKN	8.34	0.114	879	0.312	0.95	0.286	2.17	0.600	0.114	4.420	3992
CKP	4.62	0.206	1176	0.418	0.68	0.193	2.19	0.614	0.017	0.629	4533
CP	2.44	0.584	1202	0.430	1,33	0.482	2.46	0.839	0.206	0.138	4251
CPN	2.96	0.546	1062	0.377	1,36	0.512	2.44	0.864	0.182	0.125	4433
CPP	2.03	0.614	1229	0.437	1,40	0.609	2.44	0.971	0.199	0.123	4341

V_micro_: microporous volume; L_micro_: average micropore size; V_meso_: mesoporous volume by BJH method; L_meso_: average mesopore size; V_0.95_: volume until P/Po equal a 0.95; V_2_: mesoporous volume by mercury porosimetry method; V_3_: macroporous volume by mercury porosimetry method; L_macro_: average macropore size.
